# Updates on Chimeric Antigen Receptor T-Cells in Large B-Cell Lymphoma

**DOI:** 10.3390/biomedicines12122810

**Published:** 2024-12-11

**Authors:** Khalil Saleh, Nadine Khalife, Ahmadreza Arbab, Rita Khoury, Claude Chahine, Rebecca Ibrahim, Zamzam Tikriti, Nohad Masri, Mohamad Hachem, Axel Le Cesne

**Affiliations:** 1 International Department, Gustave Roussy Cancer Campus, 94800 Villejuif, France; rita.khoury011@gmail.com (R.K.); claude.chahine@gustaveroussy.fr (C.C.); rebecca.ibrahim@gustaveroussy.fr (R.I.); zamzam.tikriti@gustaveroussy.fr (Z.T.); nohad.masri@gustaveroussy.fr (N.M.); mohamadali.hachem@gustaveroussy.fr (M.H.); axel.lecesne@gustaveroussy.fr (A.L.C.); 2 Department of Head and Neck, Gustave Roussy Cancer Campus, 94800 Villejuif, France; nadine.khalife-saleh@gustaveroussy.fr; 3 Department of Biopathology, Gustave Roussy Cancer Campus, 94800 Villejuif, France; ahmareza.arbab@gustaveroussy.fr

**Keywords:** CD19-targeting CAR T-cells, lisocabtagene maraleucel, axicabtagene ciloleucel, tisagenlecleucel, large B-cell lymphoma

## Abstract

CD19-targeting chimeric antigen receptor (CAR) T-cells have changed the treatment paradigm of patients with large B-cell lymphoma (LBCL). Three CAR T-cells were approved by the Food and Drug Administration (FDA) for patients with relapsed and/or refractory (R/R) LBCL in the third-line setting: tisagenlecleucel (tisa-cel), axicabtagene ciloleucel (axi-cel), and lisocabtagene maraleucel (liso-cel), with an ORR ranging from 58% to 82%. More recently, axi-cel and liso-cel were approved as second-line treatments for patients with R/R disease up to 12 months after the completion of first-line chemo-immunotherapy. The safety profile was acceptable with cytokine release syndrome and immune effector cell-associated neurotoxicity syndrome being the two most frequent acute adverse events. Potential long-term toxicities of CD19-targeting CAR T-cells have also been described. Overall, 30% to 40% of patients are cured with a single infusion of CAR T-cells. However, 60% to 70% of patients relapse after being treated with CAR T-cells and have a dismal prognosis. The advent of bispecific antibodies (BsAb) offers an additional treatment modality for patients with R/R LBCL. The aim of this review is to describe the clinical efficacy of the three CAR T-cells, as well as their safety profile. We also compare these three CAR T-cells in terms of their efficacy and safety profile as well as evaluating the place of CAR T-cells and BsAb in the treatment arsenal of patients with R/R LBCL.

## 1. Introduction

Over the last few decades, the treatment landscape of diffuse large B-cell lymphoma (DLBCL) has changed drastically. The advent of rituximab, a CD20-targeting monoclonal antibody, has greatly improved the prognosis of patients with DLBCL [1,2]. For decades, the first-line management strategy for patients with previously untreated DLBCL was a combination of immuno-chemotherapies such as R-CHOP (rituximab, cyclophosphamide, doxorubicin, vincristine, and prednisone) or the R-ACVBP regimen (rituximab, doxorubicin, cyclophosphamide, vindesine, bleomycin, and prednisone) [1,3]. However, 30 to 40% of patients had refractory disease or relapsed after an initial response, and such symptoms are associated with a poor prognosis [4]. Autologous stem cell transplantation (ASCT) was the standard of care for patients with relapsed and/or refractory (R/R) disease preceded by salvage chemotherapy [5]. Despite these strategies, patients with R/R disease had a dismal prognosis and limited survival due to the limitations of prior strategies. Due to this unmet need, multiple efforts were made to improve the prognosis of patients with DLBCL and, notably, those with R/R disease. This has led to the development of new classes of drugs such as chimeric antigen receptor (CAR) T-cells therapy, bispecific antibodies (BsAbs), and antibody–drug conjugates (ADCs) [6]. Three CD19-targeting CAR T-cells were initially approved by the Food and Drug Administration (FDA) for the management of patients with R/R DLBCL after the failure of at least two previous treatment lines: axicabtagene ciloleucel (axi-cel), tisagenlecleucel (tisa-cel), and lisocabtagene maraleucel (liso-cel). The overall response rates (ORR) ranged from 52% to 82%. The median progression-free survival (PFS) was 2.9 months with tisa-cel, 5.8 months with axi-cel, and 6.8 months with liso-cel. The median overall survival (OS) was 12.0 months with tisa-cel, 25.8 months with axi-cel, and 27.3 months with liso-cel [7,8,9]. The FDA also approved axi-cel and liso-cel in the second-line setting in patients with R/R DLBCL who had primary refractory disease or relapsed up to 12 months after the completion of first-line immuno-chemotherapy. The approval was based on the results of the ZUMA-7 and TRANSFORM phase III trials that compared CAR T-cells with ASCT [10,11]. BsAbs are another class of drugs that are being studied more frequently and are being used to manage R/R DLBCL. To date, two drugs have been approved by the FDA: epcoritamab and glofitamab, two monoclonal antibodies targeting CD20, a B lymphocyte surface antigen, and CD3, expressed exclusively by mature T-cells [12,13]. In this review, we summarize the available clinical data of the three approved CAR T-cells as well as their safety profile. We also provide a comparison of these CAR T-cells with regard to their efficacy and safety profile and discuss their place, and that of BsAbs, in the therapeutic sequence in the management of patients with DLBCL.

## 2. CAR T-Cells Manufacturing

CAR T-cells are a synthetic product developed from an antibody sequence to bind target cell surface antigens with high specificity via a single-chain variable fragment (scFv) recognition domain [14]. This scFv domain is linked to an intracellular signaling domain derived from the CD3 zeta (CD3ζ) chain of the endogenous T-cell receptor (TCR) to stimulate the activation of T cells after antigen binding through an extracellular hinge domain and a transmembrane domain. The product is known as the first-generation CAR T-cell [15]. Multiple efforts were made thereafter to improve CAR T-cells and contribute to the production of second- and third-generation CAR T-cells by adding costimulatory signaling endodomains such as CD28 and CD137 (also known as 4-1BB) that were located between the transmembrane and CD3 signaling domains [16,17]. Full physiological T cell activation requires this costimulatory signal to be mediated by the phosphoinositide 3-kinase (PI3K) [18,19]. Moreover, fourth and fifth CAR T-cells are generated using signaling domains from cytokine receptors or the expression of inflammatory cytokines such as interleukin (IL)-12 and IL-18 [20,21]. The three CD19-targeting CAR T-cells approved in R/R LBCL are autologous, derived from the patient’s own cells; this can prevent the risk of allogenic rejection that can occur in allogenic CAR T-cells. The production of these autologous CAR T-cells begins with leukapheresis of the patient [22]. The collected T-cells are then cultivated and stimulated through CD3 and a ligand, generally CD28, in the presence of cytokines, typically IL-12 and, in some instances, IL-7 and IL-15, though this is less likely [23,24,25]. Once the T cells have been activated, the manufactured CAR is introduced into the T cells via viral or non-viral vectors [26]. CAR T-cells are then cultured in bioreactors for some days and infused into patients as a single dose. The mechanism of action of CAR T-cells starts by binding the product to a targeted antigen. In this review, CD19 expressed on the surface of the tumor cell via the scFv domain led to the activation of a killing signal of CAR T-cells, the secretion of inflammatory cytokines such as IL-2, IFN-γ, and TNF-α, and the activation of cytolytic function via perforin and granzyme [27]. The binding of CAR T-cells to the target antigen also contributed to the binding of TNF-related apoptosis-inducing ligand (TRAIL) to death receptors such as DR4 and DR5 which are expressed on the surface of tumor cells, leading to the activation of the graft versus tumor effect [28]. Moreover, the apoptosis of tumor cells could be activated via caspase 8 and the production of the death-inducing signaling complex (DISC) contributing to cell apoptosis via mature caspase 3 [29].

## 3. Clinical Efficacy of Approved CD19-Targeting CAR T-Cells

### 3.1. Axicabtagene Ciloleucel (Axi-Cel)

Axi-cel is an autologous CD19-targeting CAR T-cell with a CD28 costimulatory domain. Axi-cel was evaluated in the ZUMA-1 trial, a pivotal multicenter phase II study, in patients with R/R large B-cell lymphoma (LBCL) after receiving at least two previous lines of treatment. Axi-cel was associated with an impressive ORR of 82%, including 54% of complete remission (CR) and a median duration of response (DOR) of 11.1 months. The median PFS was 5.8 months and the median OS was 25.8 months. Nearly 93% of patients experienced cytokine release syndrome (CRS); of these patients, 13% had an AE of grade 3 or higher. The median time until the onset of CRS was 2 days after the infusion, with a median time until resolution of 8 days. Moreover, 64% of patients presented with immune effector cell-associated neurotoxicity syndrome (ICANS); for 28% of them, it was grade 3 or higher. The median time to occurrence of ICANS was 5 days after the infusion [7]. Interestingly, 31% of patients remained in CR at a median follow-up of 63.1 months suggesting that at least 30% of patients were cured with CD19-targeting CAR T-cells [30]. Based on these results, the FDA approved axi-cel as the first CD19-targeting CAR T-cell for the management of patients with R/R LBCL after failure of at least two previous lines of treatment. Moreover, retrospective real-world experience supported the findings of the ZUMA-1 trial in terms of safety and efficacy [31,32]. In addition, in the phase III ZUMA-7 trial, Axi-cel was evaluated in the second line setting in comparison with the standard of care (SOC) ASCT in patients with refractory LBCL or those with a relapse no more than 12 months after chemo-immunotherapy as first-line treatment. Axi-cel was associated with a significantly higher event-free survival (EFS) in comparison with the SOC (8.3 months versus 2.0 months, HR: 0.40; *p* < 0.001). The CR rate was 65% with axi-cel versus 35% in patients who received the SOC. Moreover, axi-cel was also associated with a significantly longer OS in comparison with SOC at a median follow-up of 47.2 months (not reached versus 31.1 months; HR, 0.73; *p* = 0.03). The safety profile was comparable to that of axi-cel in the ZUMA-1 trial. CRS of any grade occurred in 92% of patients including 6% of grade 3 or higher, while ICANS of any grade was reported in 60% of patients including 21% of grade 3 or higher [33]. In a subgroup analysis of the ZUMA-7, axi-cel was also associated with an improved OS in comparison with SOC among elderly patients ≥ 65 years and ≥70 years (43.5 months versus 19.5 months; HR, 0.69; 95% CI, 0.40–1.19; and 24.7 months versus 11.2 months; HR, 0.33; 95% CI, 0.13–0.81, respectively). Furthermore, the median PFS for axi-cel was 28.7 months versus 5.0 months for SOC in patients aged 65 years and older. These results support the consideration of CAR T-cell use in elderly patients, and particularly as second-line treatment with curative intent in patients with R/R LBCL [34]. Based on the results, axi-cel was approved by the FDA in this setting. In a real-world experience from the French Descar-T Registry of axi-cel administrated as second-line treatment, 78 patients were infused. The ORR was 77% and the CR rate was 58% among the 52 patients who had at least 1 month of follow-up. CRS was reported in 95% of patients with only less than 5% of grade ≥ 3 CRS, while neurotoxicity occurred in 43% of patients including 9% of grade ≥ 3 toxicity [35]. Moreover, axi-cel was also evaluated in the second-line setting in 62 patients with R/R LBCL who were considered ineligible for ASCT in the ALYCANTE phase II trial. The 3-month complete metabolic response (CMR) rate was 71%. The median PFS and OS were 11.8 months and not reached, respectively. The safety profile was comparable with previously reported data with axi-cel. Grade 3 or higher CRS was observed in 8.1% of patients while grade 3 or higher neurological events were reported in 14.5% of patients [36].

ZUMA-12 is a phase II trial evaluating axi-cel as first-line treatment in patients with high-risk LBCL. High-risk disease was defined as patients with MYC and BCL2 and/or BCL6 rearrangements (double or triple-hit histology) or an International Prognostic Index (IPI) score ≥ 3, plus a positive interim PET (Deauville 4/5) after 2 cycles of chemo-immunotherapy anthracyclines based. A total of 40 patients were treated with axi-cel of whom 37 patients were evaluable for response. The ORR was 92% and the CR rate was 86% at a median follow-up of 40.9 months. Interestingly, median DOR, PFS and OS were not reached and the 3-year estimated DOR, PFS and OS were 82%, 75%, and 81%, respectively. Taken together, axi-cel was associated with a high rate of durable CR with a good safety profile [37].

A pilot study of axi-cel in patients with primary or secondary central nervous system (CNS) large B-cell lymphoma (CNS LBCL) was presented at the 2024 American Society of Clinical Oncology. Overall, 18 patients were enrolled. Axi-cell showed promising results with an ORR of 94% (17/18 patients) including 67% of patients with CR, median PFS and OS were 14.3 months and 26.4 months, respectively. Interestingly, there was no grade 3 or higher CRS, but grade 3 or higher ICANS occurred in 28% of the cohort (5/18 patients) [38].

### 3.2. Tisagenlecleucel (Tisa-Cel)

Tisa-cel is an autologous CD19-targeting CAR T-cell with a 4-1BB costimulatory domain. The JULIET trial is a single-arm phase II trial evaluating the efficacy of tisa-cel in patients with R/R large B cell lymphoma (LBCL) who failed at least two previous lines of treatment. The ORR was 52% including 40% of patients achieving complete remission (CR). The median PFS and OS were 2.9 months and 12.0 months, respectively. CRS and ICANS of any grade occurred in 58% and 21% of patients, respectively. The most common grade 3 or higher adverse events (AEs) CRS in 23% of patients and ICANS in 12% of patients. The median time to the onset of CRS was 3 days, while the median to the onset of ICANS was 6 days [8]. Subsequently, and based on these results, tisa-cel was the second CD19-targeting CAR T-cell approved by the FDA as third-line treatment for patients with R/R LBCL. The results of real-world experience from the center for International Blood and Marrow transplantation Research were consistent with outcomes from the JULIET study (ORR was 59.5%) including a good safety profile with lower grade 3 or higher CRS and ICANS (6% and 7.4%, respectively) [39]. Surprisingly, in the phase III BELINDA trial, tisa-cel failed to show superiority over standard-of-care treatment in the second-line setting in patients with LBCL refractory to first-line immuno-chemotherapy or those who relapsed up to twelve months after the completion of this first-line treatment. The BELINDA trial looking at tisa-cel versus ASCT failed to meet its primary endpoint of EFS (3.0 versus 3.0 months; HR: 1.07; *p* = 0.61). Moreover, there was no OS advantage with tisa-cel in comparison with SOC (16.9 months versus 15.3 months) and the ORR was 75% with tisa-cel versus 68% with SOC. The safety profile was consistent with that reported in the JULIET trial: CRS of any grade and grade 3 or higher was reported in 61% and 5%, respectively; ICANS of any grade and grade 3 or higher occurred in 10% and 2%, respectively [40].

### 3.3. Lisocabtagene Maraleucel (Liso-Cel)

Liso-cel is the third autologous CD19-targeting CAR T-cell approved for the treatment of R/R DLBCL. Liso-cel bears a 4-1BB costimulatory domain. In contrast with tisa-cel and axi-cel, liso-cel has a different manufacturing methodology. At apheresis, CD8 and CD4 T-cells are collected and transduced separately then infused sequentially in two equal target doses [41]. Liso-cel was evaluated in the TRANSCEND-NHL-001 pivotal trial in patients with R/R LBCL who failed at least two previous lines of treatment. It is important to mention that patients with CNS involvement were allowed. The ORR was 73% including 53% of CR. The median PFS and OS were 6.8 months and 27.3 months, respectively. CRS of any grade and ICANS of any grade occurred in 42% and 30% of patients respectively. Grade 3 or higher CRS occurred in 2% of patients, while grade 3 or higher ICANS were reported in 10% of patients. The median time to the onset of CRS was 5 days, while the median time to the onset of ICANS was 9 days [9]. Based on these results, the FDA also approved liso-cel as third-line treatment for patients with R/R LBCL. In a real-world retrospective analysis from the Cell Therapy Consortium, liso-cel was associated with similar efficacy and safety profiles to that reported in prospective trials for R/R LBCL with a CR rate of 58% at 3 months reflecting a better selection of patients and improved toxicity management. At a median follow-up of 10.3 months, the 6-month PFS and OS rates were 62.7% and 77.1%, respectively [42]. Similar to the two other CAR T-cell, liso-cel was also evaluated in the second-line setting versus SOC in the phase III TRASFOM trial in patients with LBCL that relapsed or had refractory disease less than 12 months after the completion of first-line immuno-chemotherapy. The study met its primary endpoint of EFS (not reached versus 2.4 months). The CR was 74% with liso-cel versus 43% with SOC. The median OS was not reached with liso-cel versus 29.9 months with SOC. The safety profile of liso-cel was comparable to that showed in the TRANSCEND-NHL-001 trial with 49% of CRS of any grade (1% of grade 3 and higher), and 11% of ICANS of any grade (4% of grade 3 or higher) [43]. Based on these results, the FDA approved liso-cel as the second CD19-targeting CAR T-cell in this population.

Liso-cel was also evaluated in the second-line setting for the management of patients with R/R LBCL not intended for autologous stem cell transplantation in the PILOT phase II trial. Overall, 61 patients were enrolled, the ORR was 80% including 54% of CR. The median DOR was 23.3 months and was not reached in patients with CR. The median PFS and OS were 9.0 months and not reached, respectively [44].

## 4. Comparison of Approved CAR T-Cells

In the absence of randomized clinical trials that compare the efficacy and safety of the three available CD19-taregting CAR T-cells, available data comparing these agents is retrospective and comes from real-world experience or meta-analysis and matching-adjusted indirect comparison. The indirect comparison of the efficacy between the three drugs showed that axi-cel and liso-cel were associated with higher ORR (83% and 73%, respectively) than tisa-cel (52%); this is also true for the CR rate in the third-line setting [7,8,9]. Furthermore, liso-cel and axi-cel had a higher CR rate in the TRASFORM and ZUMA-7 trials (74% and 65%, respectively) than that attained by tisa-cel in the BELINDA trial (28%) [10,11,40]. Surprisingly, tisa-cel was associated with the lowest median PFS (2.9 months) among the three drugs in the third-line setting and the lowest median OS (12.0 months versus 25.8 months with axi-cel, and 27.3 months with liso-cel). In the phase III trials, tisa-cel was also associated with the lowest median EFS (3.0 months) and median OS (16.9 months). The median OS was not reached with axi-cel and liso-cel in the ZUMA-7 and TRANSFORM trials. Regarding long-term efficacy, tisa-cel was associated with 39% of CR at a median follow-up of 40.3 months, and the estimated proportion of patients with a response at 36 months was 60.4% [45]. Moreover, with a median follow-up of 63.1 months, 31% of patients treated with axi-cel had ongoing responses, and the estimated 5-year OS was 42.6%. The estimated 5-year disease-specific survival (excluding deaths unrelated to disease progression) was 51.0% [30]. However, liso-cel had a lower follow-up of 19.9 months in comparison to the two other agents. The estimated 2-year PFS and OS rates were 40.6% and 50.5%, respectively [46]. When looking at the safety profiles of the three drugs in the pivotal trials, the indirect comparison showed that axi-cel was associated with the highest rates of CRS of any grade (around 92%) and ICANS of any grade (58% to 60%) as well as grade 3 or higher CRS (6% to 13%) and ICANS (21% to 28%). However, the incidence of CRS of any grade was around 60% with tisa-cel and 42% to 49% with liso-cel. Moreover, the incidence of ICANS of any grade was 10% to 21% with tisa-cel and 11% to 30% with liso-cel [7,8,9]. Table 1 and Table 2 summarize the results of the pivotal trials of CD19-targeting CAR T-cells in third- and second-line setting.

The group of Seattle presented at the 2023 American Society of Hematology annual meeting their real-world experience with axi-cel and liso-cel. The results of 129 patients treated were reported: 81 patients received axi-cel while 48 were treated with liso-cel. In their report, axi-cel and liso-cel were associated with comparable efficacy in terms of ORR, 12-month PFS, DOR, OS rates. However, the safety profile was better with liso-cel in comparison with axi-cel with lower rates of any grade CRS and ICANS, but similar rates of grade 3 or higher CRS and ICANS [47]. These results were in line with the findings of Maloney et al. in a matching-adjusted indirect comparison of both drugs. Liso-cel had comparable ORR, OS and PFS with axi-cel but better safety profile with lower CRS and ICANS [48]. However, in another matching-adjusted indirect comparison, Oluwole and colleagues found that axi-cel was associated with significantly higher OS and PFS in comparison with liso-cel and a pejorative safety profile in terms of grade 3 or higher CRS and ICANS [49]. Moreover, in patients treated with CAR T-cells in the second-line setting, liso-cel had similar outcomes than axi-cel in terms of ORR, EFS, and PFS and better safety profile with lower rates of CRS and ICANS [50].

Axi-cel was also compared with tisa-cel in a real-world experience from the retrospective French DESCAR-T registry where 809 patients were included in the analysis. In this large retrospective cohort, axi-cel was associated with a better response with an ORR of 80% versus 66% (*p* < 0.001) and survival outcomes with 12-month PFS rate of 47% versus 33% (*p* = 0.0003) and OS of 64% versus 49% (*p* = 0.0072) in comparison with tisa-cel. However, axi-cel was associated with higher rates of CRS and ICANS than tisa-cel in patients with R/R DLBCL treated with third line or more [51]. In a recent meta-analysis, Gagelmann and colleagues confirmed the higher efficacy of axi-cel in comparison with lisa-cel with significantly improved PFS and OS and reported that axi-cel was also associated with higher any grade CRS and grade 3 of higher ICANS but not grade 3 or higher CRS [52]. However, these findings were contradictory with the results of Kwon et al. that found that axi-cel had a similar activity to tisa-cel in terms of PFS and OS but with higher toxicity in a retrospective analysis from 12 Spanish centers [53].

## 5. Management of CRS and ICANS

As previously mentioned, CRS and ICANS are the most common AEs related to CAR T-cells. CRS of any grade occurred in 42% to 93% of patients treated with approved CD19-targeting CAR T-cells, while ICANS of any grade is reported in 10% to 64% of patients. Furthermore, grade 3 or higher CRS and ICANS occurred in 1% to 22%, and 2% to 28% of patients, respectively [7,8,9]. These two AEs were initially barriers to the treatment with CAR T-cells due to deaths related to severe CRS and cerebral edema. The introduction of tocilizumab, an IL-6 receptor antagonist, and glucocorticoids led to improved management of these toxicities making CAR T-cells safer and more feasible [54]. Several grading systems for CRS had been adopted, such as the initial reports of Lee and colleagues, the grading system of the University of Pennsylvania, and the Memorial Sloan Kettering system [55,56,57]. Then came the publication of the American Society for Transplantation and Cellular Therapy (ASTCT) guidelines for CAR T-cells’ toxicity [58]. Tocilizumab remains the cornerstone of the treatment of CRS, and is recommended in patients with grade 2 CRS and in patients with grade 1 CRS with fever lasting more than 3 days according to the ASTCT [59]. Available retrospective data seem to show that the use of tocilizumab for the treatment of established CRS has no effect on the expansion of CAR T-cell or its level on the blood as well as response rate in comparison with patients who did not receive tocilizumab [31,60]. Concerning survival data, the use of tocilizumab did not seem to decrease the duration of response, PFS, or OS [32,61]. In patients with tocilizumab-refractory CRS, glucocorticoids are the standard-of-care, used with escalating doses and immediately stopping or tapering after resolution of the CRS. However, the optimal dose and schedule of steroids are not clear. The impact of glucocorticoids on efficacy and CAR T-cell persistence remains unknown. Some retrospective available data reported that the use of glucocorticoids was associated with a detrimental effect on response rates and durability of response [31]. Moreover, Strati and colleagues demonstrated that patients who received higher cumulative doses of glucocorticoids had lower PFS, and those who had prolonged use of glucocorticoids had lower OS [61]. Regarding ICANS, the ASTCT ICANS grading score is also widely used for the grading of the CAR T-cells-induced neurotoxicity. It incorporates a cognitive score, the immune effector cell-associated encephalopathy (ICE) score [58]. Glucocorticoids are the standard first-line therapy for ICANS, and once again the optimal dosing and duration are unknown. The treatment with steroids is now used for the management of grade 2 ICANS, and in some patients grade I ICANS [59]. According to the American Society of Clinical Oncology guidelines for the management of immune-related AEs after treatment with CAR T-cells, the management of ICANS with glucocorticoids might be prioritized over the treatment of low-grade CRS, given the possibility of worsening of neurological toxicity with tocilizumab [62]. Anakinra, an IL-1 receptor antagonist, is now currently used for the management of glucocorticoid-refractory ICANS [63]. Park and colleagues reported in an interim analysis from a prospective phase II trial that prophylactic anakinra resulted in a low incidence of ICANS in patients with R/R lymphoma treated with CD19-targeting CAR T-cells [64].

## 6. Long-Term Toxicities of CD19-Targeting CAR T-Cells

Initially, all studies focused on the acute toxicities of CD19-targeting CAR T-cells such as the previously described CRS and ICANS. More recently, efforts are concentrated on the identification of long-term toxicities of these drugs such as prolonged hematological toxicity (PHT), B-cell aplasia, and late infections. PHT is commonly defined as the emergence of severe anemia, neutropenia or thrombocytopenia on day 28 or 30 post infusion of CAR T-cells. It has been reported in up to 65% of patients treated with CD19-targeting CAR T-cells. The etiology of PHT remains unclear despite several mechanisms evoked such as immune-mediated hematopoietic stem cell suppression, hyperinflammatory syndrome, and transplant-associated thrombotic microangiopathy. Grade 3 or higher PHT was reported in relatively high percentage of patients after day 28. For example, neutropenia occurred in 30% to 40% of patients, thrombocytopenia in 20% to 30%, while anemia was reported in 10% to 15% of patients [65]. Cordeiro and colleagues reported that 16% of patients with ongoing CR presented PHT lasting between 15.2 and 21.7 months after the infusion of CAR T-cell [66]. Transfusion of packed red blood cells or platelets is usually necessary in patients with grade 3 or higher PHT, as well as granulocyte colony-stimulating factor that can be prescribed in patients with grade 4 neutropenia. B cell aplasia and hypogammaglobulinemia defined as IgG < 400 mg/dL were also reported in patients treated with CD19-targeting CAR T-cells. In the ZUMA-1 trial, 25% of patients had B cell aplasia and hypogammaglobulinemia at 12 months of infusion and 31% of patients received intravenous immunoglobulin (IVIG) [7]. Furthermore, in the JULIET trial, nearly 30% of patients received IVIG [8]. Late infections were also reported in patients treated with CD19-targeting CAR T-cells. Grade 3 or higher infections were reported in 8% of patients in the ZUMA-1 trial at a median of 7 to 19 months [7]. In the JULIET trial, 39% of patients treated with CD19-targeting CAR T-cells presented all-grade infection beyond 8 weeks with grade 3 or higher occurring in 18% of patients [8]. Non-relapse mortality (NRM) is another concern in patients with LBCL and treated with CD19-targeting CAR T-cells, and there is growing data regarding NRM. In a retrospective analysis from the French DESCAR-T registry sponsored by the LYSA, Lemoine and colleagues reported an estimated NRM rate of 5% (48/957 patients), the majority (81%) occurring after day 28 post infusion. Infections were the leading cause of NRM reported in 56% (of whom 27% were related to COVID-19 infection) followed by CRS in 10%, and second malignancies in 6% of cases. Interestingly, elevated ferritin level at the beginning of lymphodepletive chemotherapy and diabetes were associated with higher risk of NRM [67]. In a recent meta-analysis of patients treated with CAR T-cells for lymphoma or multiple myeloma (MM), a total of 574 non-relapse deaths were reported. Once again, infections drive NRM and were responsible for 51% of NRM followed by other malignancies (8%), and cardiac or pulmonary events (7%). However, and in contrast to Lemoine and colleagues’ paper, CRS, hemophagocytic lymphohistiocytosis, and ICANS accounted for a minority of NRM (total of 11.5%) [68]. In fact, second primary malignancies are an emerging concern following CAR T-cells. Recently, Elsallab and colleagues reported an incidence of 4.3% of second primary malignancies following CAR T-cells (536/12,394 patients). Interestingly, these cases were mainly described in patients treated with axi-cel (277/536 patients) and tisa-cel (177/536 patients). Leukemias were the most common second primary malignancies (333/536 patients) followed by skin neoplasms (54/536 patients). Leukemias were mainly myelodysplastic syndromes (208 cases), and acute myeloid leukemias (106 cases). The authors also reported 17 cases of T-cell non-Hodgkin lymphoma representing only 0.1% of all second primary malignancies following CAR T-cells [69]. The FDA announced T cell malignancies following treatment with CAR T-cells either CD19-targeting or BCMA-targeting drugs. This alert was based on initial reports of 22 cases of T-cell lymphoma including three patients with lymphomas containing CAR viral vectors [70]. The University of Stanford analyzed 724 patients treated with adoptive cellular therapies. The incidence of second primary malignancies was 3.5% (25/724 patients) excluding nonmelanoma skin cancers and only one case of T-cell lymphoma [71]. These findings were also concordant with the reports of the University of Pennsylvania with 3.6% of second primary malignancies (16/449 patients) with also one case of T-cell lymphoma [72]. Overall, there is no data to identify CAR T-cells as drivers for the second malignancies.

## 7. Mechanisms of Resistance to CAR T-Cells

Despite the interesting activity of CD19-targeting CAR T-cells in the management of patients with R/R LBCL, only 30% to 40% of patients are cured with these drugs. The efficacy is counterbalanced by the emergence of primary or secondary (acquired) resistance. In the registration ZUMA-1, JULIET, and TRANSCEND-NHL-001 trials in third line setting or more, the CR rates ranged between 40% and 58% suggesting that primary resistance existed in nearly half of patients with R/R LBCL treated with CD19-taregting CAR T-cells (60% of patients treated with tisa-cel, 42% of patients treated with axi-cel, and 47% of patients who received liso-cel). Secondary resistance is defined by the emergence of resistance in patients who already achieved a CR following treatment with CD19-targeting CAR T-cells. It has been suggested that patients with low burden of disease presented higher efficacy and decreased toxicity [73]. Moreover, worse ECOG performance status, higher stage of disease, more than 2 extranodal sites, elevated international prognostic index, and high lactate dehydrogenase and/or C-reactive protein were defined as clinical risk factors for disease recurrence [74]. Several biologic mechanisms of resistance to CAR T-cells had been evoked such as CAR T-cell dysfunction, tumor-intrinsic resistance, as well as immunosuppressive tumor microenvironment (TME). CAR T-cell dysfunction might be related to the exhaustion of CAR T-cell that could be seen in case of persistent antigen stimulation [75]. The presence of immunosuppressive cells and the expression of inhibitory receptors such as cytotoxic T lymphocyte antigen 4 (CTLA-4), lymphocyte activation gene 3 (LAG3), and programmed cell death 1 (PD1) led also to CAR T-cell dysfunction, decreased cytokine production, decreased cytotoxicity, and reduced proliferative capacity [76,77]. Tumor-intrinsic resistance is mainly related to the absence of antigen on tumor cells or to truncated antigen in case of point mutations or alternative splicing. It could also be related to tumor resistance to apoptosis by downregulation of receptors or overexpression of anti-apoptotic proteins. The role of TME is also important in the prediction of response to CAR T-cells. The presence of barriers that inhibit CAR T-cells infiltration is a mechanism of resistance and leads to dysregulated adhesion and vascular permeability. The presence of tumor-associated macrophages (TAM) and regulatory T cells (Treg) and immunosuppressive cytokines such VEGF, TGFbeta, and IL10 could also confer resistance to CAR T-cells [78].

## 8. CAR T-Cells or Bispecific Antibodies

As previously mentioned, the advent of BsAbs in the last decade has also revolutionized the treatment of patients with R/R LBCL. BsAbs are genetically produced to recognize and bind to two different targets such as epitopes or antigens simultaneously. Two monospecific antigen-binding regions, which freely recognize their respective precise target, are combined to make a single antibody-derived molecule that acts as a bridge for these two antigens [6]. In contrast to CAR T-cells that need leukapheresis ex-vivo transduction of autologous T cells, and manufacturing of CAR T-cells before the infusion of the drug—a process that takes 2 to 4 weeks, BsAbs are an off-the-shelf way to stimulate the body-s T-cell immune system to fight B-cell lymphoma and can be used immediately without manufacturing process [79]. As previously mentioned, two BsAbs (epcoritamab and glofitamab) had been approved for the management of patients with R/R LBCL. In the EPCORE NHL-1, a phase I/II trial evaluating epcoritamab, a BsAb targeting CD20 and CD3, 39% of enrolled patients previously received CAR T-cells. At a median follow-up of 10.7 months, the ORR in the entire cohort was 63% including 39% of CR. The median DOR was 12.0 months and was not reached among responders. The median PFS was 4.4 months, while the median OS was not reached. The safety profile of epcoritamab is acceptable. CRS of any grade was reported in 49.7% of patients including 2.5% of grade 3, while ICANS of any grade grade occurred in 6.4% of patients including only one grade 3 (0.6%) [12]. Furthermore, glofitamab another BsAb targeting CD20 and CD3, were evaluated in a phase I/II trial in patients with R/R DLBCL. In the entire cohort, glofitamab was associated with an ORR of 53%, and a CR rate of 39% at a median follow-up of 12.6 months. The median PFS was 4.9 months, and the 12-month OS rate was 50%. Regarding safety profile, CRS of any grade was reported in 63% of patients including 4% of grade 3 or higher. ICANS was observed in only 8% of patients including 3% of grade 3 or higher [13]. Prospective trials of BsAbs demonstrated that patients with R/R LBCL who failed CD19-targeting CAR T-cells responded also to BsAbs. In the EPCORE NHL-1, 39% of enrolled patients were previously received CAR T-cells. Interestingly, in patients previously treated with CAR T-cells the ORR was 54% with 34% of CR [12]. Furthermore, Dickinson and colleagues found that patients who previously failed CAR T-cells and treated with glofitamab (one third of patients) had similar CR rate in comparison with those who had not received CAR T-cells (35% versus 42%, respectively) in patients with R/R DLBCL [13]. In the other way, available retrospective data suggest that CAR T-cells remain effective in patients with LBCL previously treated with BsAbs. However, prospective evidence of efficacy has not been published yet. Crochet and colleagues reported a retrospective analysis of 47 patients who received CD19-targeting CAR T-cells and previously treated with BsAbs. In total, 83% of patients infused showed response to CAR T-cell with a median PFS of 6.6 months and the median OS was not reached. This cohort was matched with another cohort of patients who did not receive BsAbs before CAR T-cells with a propensity score. The 2 cohorts shared the same PFS, OS and toxicities suggesting that treatment with BsAbs has no impact on the efficacy of subsequent CAR T-cells [80]. Moreover, preclinical data suggest that the co-administration of CD20-directed BsAbs with CD19-targeting CAR T-cells resulted in enhanced treatment efficacy and increased elimination of tumoral cells. This co-administration resulted also in prolonged survival [81].

More recently, Kim and colleagues published a meta-analysis comparing CD19-targeting CAR T-cells and BsAbs in patients with LBCL treated in third-line or later-line setting. In this meta-analysis, CD19-targeting CAR T-cells were associated with a higher efficacy with a pooled CR rate of 0.51 with CAR T-cells versus 0.36 with BsAbs (*p* < 0.01). This higher efficacy persisted even in patients naïve to CAR T-cells and treated with BsAbs, with a CR rate of 0.37. Furthermore, the pooled 12-month PFS rate was also significantly higher in the CAR T-cells group in comparison with the BsAbs group (0.44 versus 0.32; *p* < 0.01). As expected, treatment with CD19-targeting CAR T-cells was associated with a higher incidence of grade 3 or higher AEs, with regards to CRS and neurotoxicity, in comparison to patients treated with BsAbs. However, the incidence of grade 3 or higher infections was similar between the two groups [82]. These results were also in line with findings reported in patients with follicular lymphoma. Recently, a match-adjusted indirect comparison of liso-cel and mosunetuzumab, a BsAb targeting CD20 and CD3 approved for the treatment of follicular lymphoma (FL), in the treatment of patients with R/R FL in third line and beyond shows similar results. In this comparison, liso-cel was found to have a higher efficacy than mosunetuzumab in terms of ORR, CR rate, and PFS. Regarding the safety profile, liso-cel was associated with a higher proportion of any-grade CRS, ICANS and tocilizumab use for CRS, while mosunetuzumab showed a higher incidence of grade 3 or more CRS, ICANS, and steroid use for management of CRS [83]. Taken together, these data support the suggestion of starting with CAR T-cells in the management of R/R LBCL followed by BsAbs in case of disease relapse.

This strategy could change in case BsAbs move into first-line treatment of patients with newly diagnosed LBCL. The EPCORE-NHL2 is a phase I/II trial evaluating epcoritamab, a BsAB targeting CD20 and CD3, in combination with RCHOP in patients with newly diagnosed high-risk DLBCL. A total of 47 patients were treated. The combination was associated with impressive results. The ORR was 100% in evaluable patients (46/46) with complete metabolic response (CMR) in 76% of patients (35/46) with an acceptable safety profile. Interestingly, at a median follow-up of 11.5 months, the median OS, PFS and DOR were not reached [84]. These data support the ongoing EPCORE DLBCL-2 phase III trial comparing epcoritamab plus R-CHOP versus R-CHOP in patients with newly diagnosed DLBCL [85]. The results of EPCORE-NHL2 were consistent with the results NP40126 trial phase Ib trial evaluating glofitamab, another BsAb targeting CD20 and CD3, in combination with R-CHOP in patients with previously untreated DLBCL. Once again, the combination of BsAb with immune-chemotherapy was highly effective with an ORR of 93% (52/56) and a CMR of 84% (47/56) with a manageable safety profile with low incidence of CRS [86].

## 9. Conclusions and Future Perspectives

In conclusion, CD19-targeting CAR T-cells changed the treatment landscape of patients with R/R LBCL. Three drugs are approved by the FDA in the third-line setting (axi-cel, tisa-cel, and liso-cel). Axi-cel and liso-cel are also approved in the second-line setting in patients with refractory disease or relapse up to 12 months after the completion of first-line chemo-immunotherapy. These drugs are associated with an acceptable safety profile and two main acute toxicities (CRS and ICANS). Long-term toxicities also occur with CAR T-cells such as B-cell aplasia and late onset infections. The efficacy of CD19-targeting CAR T-cells is counterbalanced by the emergence of resistance. Several strategies are currently under evaluation in order to improve outcomes of treatment with CAR T-cells in patients with R/R LBCL. Table 3 and Table 4 summarize the major ongoing clinical trials of axi-cel and liso-cel in patients with R/R LBCL. Sequential treatment with CD19-targeting CAR T-cells followed by BsAbs such as mosunetuzumab or glofitamab is under evaluation in patients with R/R DLBCL or transformed FL in a phase II trial (NCT04889716). Moreover, another strategy is based on a consolidation treatment in patients who are not in CR after CD19-targeting CAR T-cells such as consolidation with epcoritamab in a randomized phase II trial (NCT06238648) or loncastuximab tesirine, an antibody-drug conjugate (ADC) approved for the treatment of R/R LBCL, in a phase II trial (NCT05464719). Bridging treatment with glofitamab in combination with rituximab plus ifosfamide, carboplatin, etoposide in participants with R/R transplant or CAR-T therapy eligible DLBCL is also under evaluation (NCT05364424). Another trial, the PORTAL trial, is also evaluating polatuzumab vedotin, obinutuzumab and glofitamab as a peri-CAR T-Cell treatment strategy in LBCL (NCT06071871). Table 3 and Table 4 summarize the major trials evaluating axi-cel and liso-cel in patients with R/R LBCL.

## Figures and Tables

**Table 1:** Three major trials of CD19-targeting CAR T-cells in third-line setting.

Study	Agent	Nb of Patients	Nb of Infused Pts	ORR/CR Rate	Median PFS (Months)	Median OS (Months)	CRS All Grade/Grade ≥ 3	ICANS All Grade/Grade ≥ 3
ZUMA-1 [7,30]	Axi-cel	111	101 (91%)	83%/58%	5.8	25.8	93%/13%	64%/28%
JULIET [8]	Tisa-cel	165	111 (67%)	52%/40%	2.9	12.0	58%/22%	21%/12%
TRANSCEND-NHL-001 [9,46]	Liso-cel	344	269 (78%)	73%/53%	6.8	27.3	42%/2%	30%/10%

ORR: overall response rate; CR: complete remission; PFS: progression free-survival; OS: overall survival; CRS: cytokine release syndrome; ICANS: immune effector cell-associated neurotoxicity syndrome.

**Table 2:** Three major phase III trials in second line in R/R LBCL.

Study	Agent	Nb of Patients	Median Follow-Up (Months)	CR Rate	Median EFS (Months)	Median OS (Months)	CRS All Grade/Grade ≥ 3	ICANS All Grade/Grade ≥ 3
ZUMA-7 [10,33]	Axi-cel vs. SOC	359	47.2	65% vs. 32%	10.8 vs. 2.3	NR vs. 31.1	92%/6%	60%/21%
BELINDA [40]	Tisa-cel vs. SOC	322	10	28% vs. 28%	3.0 vs. 2.0	16.9 vs. 15.3	61%/5%	10%/2%
TRANSFORM [11,43]	Liso-cel vs. SOC	184	17.5	74% vs. 43%	NR vs. 2.4	NR vs. 29.9	49%/1%	11%/4%

CR: complete response; SOC: standard-of-care; EFS: event-free survival; OS: overall survival; CRS: cytokine release syndrome; ICANS: immune effector cell-associated neurotoxicity syndrome.

**Table 3:** Major ongoing trials evaluating axi-cel in patients with LBCL.

Study	Drugs	Phase	No of Patients	Population	Primary Endpoint
NCT05794958	Axi-cel	Ib	20	Reinfusion in R/R second-line high-risk LBCL after SOC axi-cel	DLTs
NCT04257578	Axi-cel + acalabrutinib	I/II	50	LBCL	AEs
NCT05459571 (ZUMA-24)	Axi-cel + steroids	II	30	R/R LBCL	TE-CRS, TENE
NCT05605899 (ZUMA-23)	Axi-cel vs. SOC	III	300	First-line treatment high-risk LBCL	EFS
NCT06213311	Axi-cel + glofitamab	II	40	Second-line R/R LBCL	AEs
NCT05757219	Itacitinib pre axi-cel	II	27	DLBCL	PFS

TE-CRS: treatment emergent cytokine release syndrome; TENE: treatment emergent neurological events; LBCL: large B cell lymphoma; R/R: relapsed and/or refractory; SOC: standard-of-care; AEs: adverse events; EFS: event-free survival; PFS: progression-free survival; DLTs: dose-limiting toxicities.

**Table 4:** Major ongoing trials of liso-cel in patients with LBCL.

Study	Drugs	Phase	No of Patients	Population	Primary Endpoint
NCT05583149	Acalabrutinib + liso-cel	II	27	R/R aggressive B-cell lymphoma after 2 lines	CR rate
NCT05873712	Zanubrutinib + liso-cel	II	24	R/R richter’s syndrome	ORR
NCT05672173	Liso-cel + nivolumab + ibrutinib	II	20	R/R richter’s syndrome	CR UT
NCT05359211	Liso-cel + NKTR-255	I	24	R/R LBCL	AEs DLT CR rate
NCT05664217	NKTR-255 vs. placebo after liso-cel	II/III	400	R/R LBCL	EFS CR rate

UT: unacceptable toxicity; CR: complete remission; ORR: objective response rate; AEs: adverse events; DLT: dose-limiting toxicities; EFS: event-free survival; R/R: relapsed and/or refractory; LBCL: large B cell lymphoma.
